# Supplementary material for: Diagnostic and Prognostic Nomograms for Hepatocellular Carcinoma Based on PIVKA-II and Serum Biomarkers
Source: Diagnostics (Basel). 2023 Apr 17;13(8):1442. doi: 10.3390/diagnostics13081442 (PMC10138067; doi:10.3390/diagnostics13081442)
Supplement: Supplementary file 1 [file diagnostics-13-01442-s001.zip › diagnostics-2258595-supplementary.pdf]

diagnostics

Supplementary Materials

Table S1. Differences in the rates of HCC diagnosis between AFP and PVIKA-II.

		AFP		Total
		Positive	Negative	
PVIKA-II	Positive	197	100	297 (84.37%)
	Negative	30	25	55 (15.63%)
Total		227 (64.49%)	125 (35.51%)	352

Table S2. Characteristics of the patients in the training cohort and validation cohort.

Parameters	Training cohort (n=142)	Validation cohort (n=99)	*P* value
Age (years) Median (IQR)	54.0(48.0-64.0)	55.0 (47.0-63.5)	0.936[a]
Gender Male (%)	122 (85.9%)	88 (88.9%)	0.498[b]
Child-Pugh grade			0.401[b]
A	114 (80.3%)	75 (75.8%)	
B	28 (19.7%)	24 (24.2%)	
BCLC			0.499[b]
A	29 (20.4%)	16 (16.2%)	
B	74 (52.1%)	59 (59.6%)	
C	39 (27.5%)	24 (24.2%)	
AFP (mg/mL) Median (IQR)	49.5 (5.7-1090.9)	134.050 (14.5-2814.0)	0.341[a]
PVIKA-II (mAU/mL) Median (IQR)	486.8 (64.3-3442.3)	818.1 (93.4-5834.1)	0.174[a]
CEA (mg/mL) Median (IQR)	2.4 (1.6-3.9)	2.875 (1.433-4.327)	0.454[a]
CA199 (mg/mL) Median (IQR)	8.5 (5.0-19.1)	10.8 (4.2-18.7)	0.997[a]
WBC ($\times 10^9$/L) Median (IQR)	7.610(5.140-10.170)	7.7 (5.5-9.5)	0.858[a]
LY ($\times 10^9$/L) Median (IQR)	1.1 (0.7-1.6)	1.1 (0.7-1.6)	0.752[a]
NET ($\times 10^9$/L) Median (IQR)	5.1 (3.1-7.9)	4.8 (3.4-7.9)	0.928[a]

NLR<br>Median (IQR)	4.5 (2.8-8.0)	4.6 (2.6-9.1)	0.664[a]
RBC (×10^9/L)<br>Median (IQR)	4.1 (3.4-4.5)	4.0 (3.4-4.4)	0.284[a]
Hb (g/L)<br>Median (IQR)	132.5 (122.3-144.0)	126.0 (110.0-143.0)	0.081[a]
RDW (%)<br>Median (IQR)	14.0 (13.0-15.0)	13.0 (12.2-15.0)	0.287[a]
PLT (×10^9/L)<br>Median (IQR)	165.0 (101.0-223.0)	166.0 (108.0-223.5)	0.954[a]
MPV (fL)<br>Median (IQR)	10.0 (9.1-10.9)	10.0 (9.3-11.0)	0.519[a]
ALT (U/L)<br>Median (IQR)	41.0 (23.2-63.0)	42.0 (30.0-78.0)	0.193[a]
AST (U/L)<br>Median (IQR)	45.0 (30.2-71.0)	48.0 (37.0-96.5)	0.099[a]
GGT<br>Median (IQR)	76.0 (49.8-146.3)	87.5 (46.3-218.8)	0.396[a]
LDH<br>Median (IQR)	232.5 (198.5-311.0)	239.5 (198.0-322.8)	0.982[a]
TBIL (μmol/L)<br>Median (IQR)	18.3 (13.7-24.8)	19.0 (12.5-28.7)	0.953[a]
DBIL (μmol/L)<br>Median (IQR)	4.4 (2.8-8.2)	4.2 (2.9-8.8)	0.895[a]
ALB (g/L)<br>Median (IQR)	36.8 (33.4-40.2)	36.0 (32.3-40.1)	0.374[a]
TP (g/L)<br>Median (IQR)	67.5 (62.7-71.4)	65.0 (59.7-71.3)	0.080[a]
Cr (μmol/L)<br>Median (IQR)	68.0 (56.3-77.0)	70.0 (59.8-85.5)	0.112[a]
PT (s)<br>Median (IQR)	12.5 (11.7-13.5)	12.7 (12.0-14.3)	0.007[a]
APTT (s)<br>Median (IQR)	30.1 (27.2-35.3)	29.2 (27.2-33.0)	0.120[a]
TT (s)<br>Median (IQR)	18.1 (17.2-19.1)	17.9 (17.2-19.1)	0.316[a]
FIB (g/L)<br>Median (IQR)	2.7 (2.1-3.6)	2.9 (2.1-3.5)	0.705[a]

Categorical and continuous variables are presented as frequencies or medians (IQR), respectively. Differences between groups in categorical and continuous variables are analyzed using the chi-squared test and Mann-Whitney U test, respectively.

BCLC: Barcelona Clinic Liver Cancer; HBsAg: HBV surface antigen; CEA: Carcinoembryonic antigen; CA199: Carbohydrate antigen 199; LY: Lymphocyte; NET: Neutrophils; NLR: Neutrophil-to-lymphocyte ratio; RDW: Red cell distribution width; MPV: Mean Platelet Volume; Cr: Creatinine; TBIL: Total bilirubin; DBIL: Direct bilirubin; INR: International Normalized Ratio; APTT: Activated

partial thromboplastin time; TT: Thrombin time; FIB: Fibrinogen.
[a] Wilcoxon rank sum test; [b] Chi-squared test.
